# Mortality due to primary brain tumours in China and detection rate in people with suspected symptoms: a nationally representative cross-sectional survey

**DOI:** 10.1186/s12957-021-02179-5

**Published:** 2021-03-12

**Authors:** Bin Jiang, Hongmei Liu, Dongling Sun, Haixin Sun, Xiaojuan Ru, Jie Fu, Siqi Ge, Wenzhi Wang

**Affiliations:** 1 Department of Neuroepidemiology, Beijing Neurosurgical Institute, Beijing Tiantan Hospital, Capital Medical University, No. 119, South Fourth Ring Road West, Fengtai District, Beijing, 100070 People’s Republic of China; 2 Beijing Municipal Key Laboratory of Clinical Epidemiology, Beijing, People’s Republic of China; 3 National Office for Cerebrovascular Diseases (CVD) Prevention and Control in China, Beijing, People’s Republic of China

**Keywords:** Epidemiology, Mortality, Detection rate, Primary brain tumours, China

## Abstract

**Background and purpose:**

Epidemiological data on primary brain tumours (PBTs) are lacking due to the difficulty in case ascertainment among the population. Thus, we aimed to estimate mortality due to PBTs in China nationwide and the detection rate in people with suspected symptoms.

**Methods:**

A multistage, complex sampling survey regarding mortality due to PBTs in Chinese individuals was carried out by reviewing all causes of death within a year. The detection rates in people with suspected symptoms were estimated based on PBT symptom screening and neurologist reviews and compared between groups by logistic regression analysis.

**Results:**

Weighted mortality due to PBT was 1.6 (0.8–3.3) per 100,000 population in Chinese individuals, 1.8 (0.7–4.6) per 100,000 population in men, and 1.5 (0.5–4.5) per 100,000 population in women. Among 14,990 people with suspected symptoms, the PBT detection rate was 306.9 (95% CI 224.7–409.3) per 100,000 population in the total population, 233.0 (95% CI 135.7–373.1) per 100,000 population in men, and 376.9 (95% CI 252.4–546.3) per 100,000 population in women. People with an unsteady gait (OR 2.46; 95% CI 1.09–5.51; *P*=0.029), visual anomalies (3.84; 1.88–7.85; *P*<0.001), and headache (2.06; 1.10–3.86; *P*=0.023) were more likely to have a brain tumour than those without corresponding symptoms, while people with dizziness/vertigo were less likely to have a brain tumour than those without corresponding symptoms (0.45; 0.23–0.87; *P*=0.017).

**Conclusions:**

Mortality due to PBT in China was low, with a nationwide estimate of 21,215 (10,427–43,165) deaths attributable to PBTs annually. However, the detection rate of PBTs can be greatly improved based on symptom screening in the population.

## Introduction

Primary brain tumours (PBTs) are characterized by high rates of case fatality and disability [1]. However, epidemiological data on PBTs are lacking due to difficulty in brain tumour case ascertainment among people without specific symptoms in the population. In the USA [2, 3] and Europe [4], the prevalence of primary brain tumour in the population can be estimated from brain tumour-related incidence and survival data. There are three major symptoms of brain tumour: headache, vomiting, and blurred visions due to optic disc oedema, and motor and sensory dysfunctions. Although these symptoms are nonspecific, it seems to be feasible to investigate individuals’ symptoms in the population. Thus, we adopted a multistage, complex sampling method to investigate mortality due to PBTs in China and the detection rate in people with suspected symptoms (/signs) by innovatively establishing a two-step method, including screening for common central nervous system symptoms that overlap with stroke by staff from China’s Center for Disease Control and Prevention and differentiating brain tumour and stroke cases by neurologists based on the national epidemiological survey of cerebrovascular diseases in China [5–9].

## Methods

### Sampling and participants

**Fig. 1:**
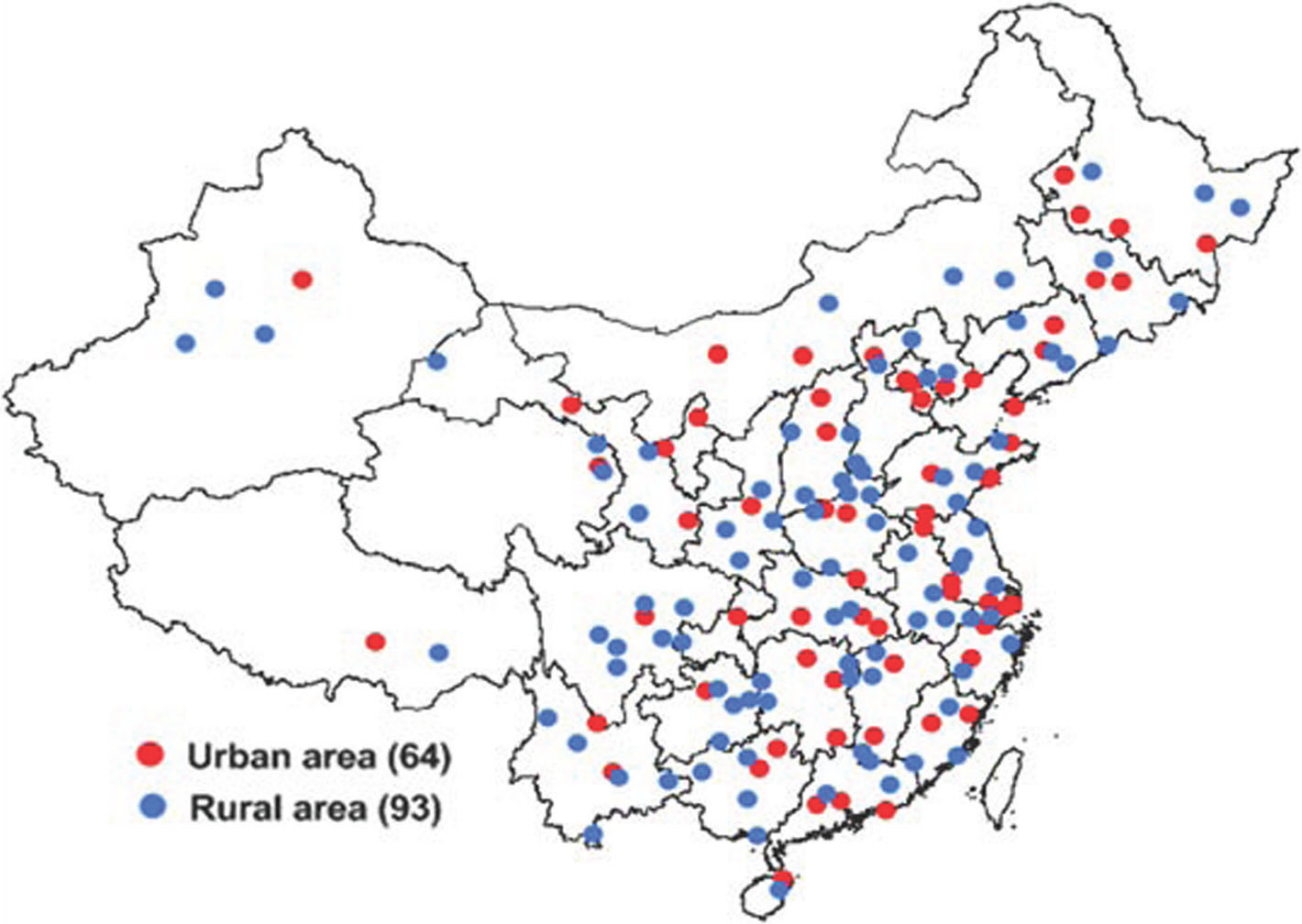
Distribution of survey sites in 31 provinces of China

The complex, multistage probability sampling design used to define the sampling frame and the participants has been described in detail in previous studies [5–7]. In brief, 2010 Chinese population census data and probability proportionate to population size (PPS) sampling were used to select 64 urban and 93 rural areas from 31 provinces of China [i.e., 157 disease surveillance points (DSPs) or survey sites shown in Fig. 1]. In the first stage of sampling, PPS sampling was again used to select ‘neighbourhoods’ (Jiedao) within cities or ‘townships’ (Xiang) in rural areas; the probability of selection was based on the population size of the neighbourhood or township. In the second stage of sampling, one or more neighbourhood committees (administrative villages) with a total population of at least 4500 residents (approximately 1500 households) were selected from the sampled neighbourhoods (townships) at each site using random cluster sampling. The participants included people who had lived in the county (or district) for at least 6 months in the past year. Trained investigators visited these participants at least 3 times on different dates. For this survey, 602,715 people in 155 DSPs were evaluated, with a response rate of 80.8% among 745,588 people [6].

### Case ascertainment and quality assurance

**Fig. 2:**
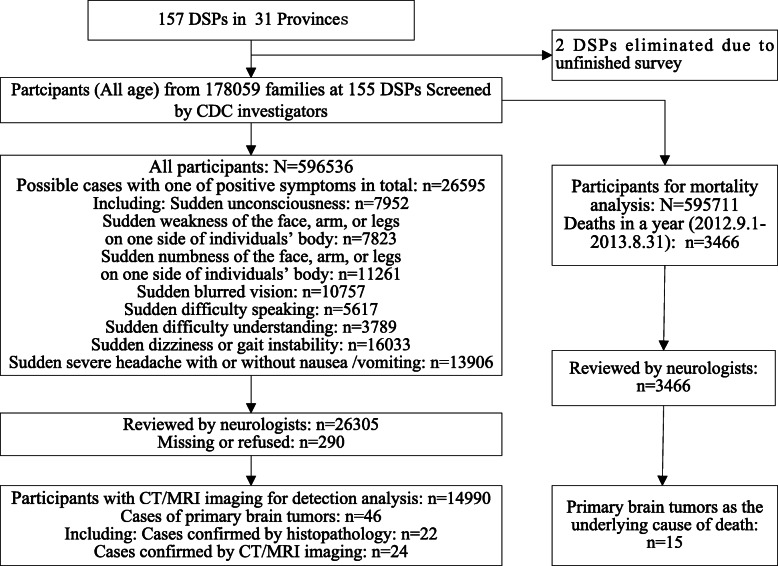
Flowchart of primary brain tumour case ascertainment. Note: DSP, disease surveillance points; CDC, Center for Disease Control and Prevention

The national survey on brain tumours and stroke was conducted with face-to-face household interviews. From September 1, 2013, through December 31, 2013, CDC investigators visited each household, collected participants’ signed informed consent forms and completed the preliminary screening form. This questionnaire included basic information about family members, symptoms, and the medical history of the individual. In this survey, participants with at least one of eight symptoms (*n*=26595) including sudden unconsciousness (*n*=7952); weakness in the face, arms, or legs on one side of the body (*n*=7823); numbness in the face, arms, or legs on one side of the body (*n*=11261); blurred vision (*n*=10757); difficulty speaking (*n*=5617); difficulty understanding (*n*=3789); dizziness or gait instability (*n*=16033); or severe headache with or without nausea/vomiting (*n*=13906) were identified by China’s Center for Disease Control and Prevention (CDC) investigators and subsequently interviewed by neurologists. After preliminary screening, participants with symptoms or history suggestive of brain tumour were invited to see a neurologist in a town/village clinic. Their medical records [e.g., disease history, head computed tomography (CT) and magnetic resonance imaging (MRI) scans] were carefully reviewed, and relevant data were recorded. At the review/confirmation stage of the survey, neurologists interviewed 26,305 participants with one of the abovementioned eight symptoms and completed relevant case adjudication forms. Neurological examinations and reviews were completed for 98.9% of the eligible patients. Among these study participants, 14,990 suspected cases had CT/MRI or histopathological confirmation (Fig. 2). The validated verbal autopsy technique involving household members of people who died within the 12 months preceding the survey was used to identify PBT as a possible cause of death. Ultimately, 46 surviving patients and 15 decedents were diagnosed with PBT by neurologists (Fig. 2).

Detailed quality assurance methods have been described in previous studies [5–7]. In brief, quality control was performed in all phases of the survey, and survey preparations, field work, and data processing were all supervised.

### Diagnostic criteria

PBTs were defined as tumours originating from the brain, brain stem, meninges, cranial nerves, and other parts of the brain or the intracranial endocrine glands. Tumours involving only the spinal cord were not included. The diagnostic criteria for PBT in this study were as follows: (1) typical history and (/or) symptoms of intracranial tumour; (2) CT/MRI confirmation; (3) diagnosis by a neurosurgeon (based on clinical and radiological examinations) and (/or) surgical and pathological confirmation (with diagnostic proof); (4) neurologist consultation for diagnosis confirmation; and (5) the exclusion of brain metastasis. All the tumours diagnosed in this study were confirmed by hospital examination or operation [10, 11]. All PBT cases resulting in death were identified based on previous medical histories and death certificates according to the International Classification of Diseases, Tenth Revision, Clinical Modification (ICD-10-CM).

### Statistical analysis

Statistical analyses were conducted with weighted data to account for the complex sampling design. Weighted coefficients were calculated by considering the sampling weights, nonresponse weights, and poststratification weights to obtain the national estimates. Population data from the 2010 Chinese population census were used to calculate poststratification weights adjusted for sex (men, women), age (0–4, 5–9, 10–14, 15–19, 20–24, 25–29, 30–34, 35–39, 40–44, 45–49, 50–54, 55–59, 60–64, 65–69, 70–74, 75–79, 80+ years), residence (urban or rural), and geographic location (eastern, central, or western China) [6, 9].

In this retrospective epidemiological survey, mortality due to PBT was defined as the rate of the dead cases with PBT within a year among the survival population prior to midnight on August 31, 2012, from the sampled families. National estimates of mortality and 95% confidence intervals (CIs) were computed for age and sex groups with final weights.

Similarly, the detection rate of PBTs was defined as the rate of PBT cases confirmed by CT/MRI detection or histopathology among survivors with suspected symptoms (/signs) from the sampled families prior to midnight on August 31, 2013. Crude detection rates and 95% CIs were estimated with Poisson distribution, because the small counts of brain tumour have the distribution.

Detection rates among people with suspected symptoms (/signs) were calculated and further compared between subgroups in the multivariate analysis. Factors influencing the detection rates of primary brain tumour in participants with suspected symptoms (/signs) were determined after adjustment for different explanatory factors in a binary logistic regression model. The explanatory risk factors included age group (0–14, 15–24, 25–34, 35–44, 45–54, 55–64, 65–74, ≥ 75 years); sex (men/women); ethnicity (Han ethnicity/other ethnicity); education (primary school/middle school/college and higher/missing); marriage status (married/single/widowed/other/missing); occupation (students/worker/farmer/employee/entrepreneur/retiree or homemaker/other/missing); place of residence (urban/rural); geographic location (eastern/central/western China); disturbance of consciousness (yes/no); limb paralysis or weakness (yes/no); facial paralysis (yes/no); gait instability (yes/no); speech disorders (yes/no); paraesthesia (yes/no); visual anomalies (yes/no); dizziness/vertigo (yes/no); headache (yes/no); and nausea/vomiting (yes/no).

All of the statistical calculations were performed with SPSS 15.0 software (SPSS Inc., Chicago, IL, USA). *P* <0.05 was considered statistically significant.

## Results

### Characteristics of the participants and patients with PBTs

The characteristics of the study sample from the national epidemiological survey of PBTs in China 2013 are shown in Table 1. Among the 595,711 people included in the mortality analysis, 15 of 3466 deaths occurring between September 1, 2012, and August 31, 2013, were due to PBTs (see Fig. 2 and Table 2). Among the 14,990 people evaluated in the detection rate analysis, 46 survivors with PBT confirmed by CT/MRI or histopathology were identified on August 31, 2013 (see Fig. 2 and Table 3). There were 2 glioma, 7 pituitary adenoma, 7 meningioma, 3 acoustic neuroma, 1 craniopharyngioma, 1 cerebellar haemangioblastoma, 1 primary central nervous system lymphoma (PCNSL), and 24 undetermined intracranial tumour cases among the 46 cases; 6.5% experienced conscious disturbance, 28.3% experienced limb paralysis or weakness, 4.3% experienced facial paralysis, 30.4% had an unsteady gait, 13.0% had speech disorders, 17.4% experienced paraesthesia, 26.1% had visual anomalies, 37.0% had dizziness/vertigo, 47.8% had headache, and 15.2% had nausea/vomiting.

**Table 1:** Characteristics of the study sample from the national epidemiological survey of primary brain tumours in China, 2013

	Sample for mortality			Sample for detection rate	
Characteristic	No.	%	Weighted^a^ %	No.	%
Age group					
0~	85,462	14.3%	17.4%	28	0.2%
15~	81,378	13.7%	17.3%	34	0.2%
25~	89,597	15.0%	15.1%	143	1.0%
35~	102,999	17.3%	18.5%	560	3.7%
45~	90,667	15.2%	13.4%	1917	12.8%
55~	78,075	13.1%	10.2%	4767	31.8%
65~	43,245	7.3%	5.3%	4590	30.6%
75~	24,288	4.1%	2.9%	2951	19.7%
Sex					
Men	299,725	50.3%	51.1%	7296	48.7%
Women	295,986	49.7%	48.9%	7694	51.3%
Ethnicity					
Han	518,151	87.0%	91.4%	13,756	91.8%
Other	77,560	13.0%	8.6%	1234	8.2%
Education, *n* (%)					
Primary school	245,192	41.2%	38.9%	9176	61.2%
Middle school	294,193	49.4%	49.2%	5297	35.3%
College and higher	51,721	8.7%	11.3%	504	3.4%
Missing	4605	0.8%	0.5%	13	0.1%
Marital status, *n* (%)					
Married	391,124	65.7%	61.2%	12,059	80.4%
Single	115,438	19.4%	23.8%	238	1.6%
Widowed	32,731	5.5%	4.2%	2640	17.6%
other	51,402	8.6%	10.2%	33	0.3%
Missing	5016	0.8%	0.6%	20	0.1%
Occupation, *n* (%)					
Students	105,510	17.7%	22.4%	42	0.3%
Worker	45,004	7.6%	8.8%	620	4.1%
Farmer	270,916	45.5%	38.8%	8343	55.7%
Employee	46,674	7.8%	9.9%	350	2.3%
Entrepreneur	52,516	8.8%	10.2%	393	2.6%
Retiree or homemaker	66,145	11.1%	8.7%	5154	34.4%
other	4356	0.7%	0.7%	76	0.5%
Missing	4590	0.8%	0.5%	12	0.1%
Place of residence					
Urban	282,169	47.4%	52.8%	7628	50.9%
Rural	313,542	52.6%	47.2%	7362	49.1%
Geographic location					
Eastern China	201,196	33.8%	40.7%	4950	33.0%
Central China	239,288	40.2%	32.0%	7073	47.2%
Western China	155,227	26.1%	27.3%	2967	19.8%

^a^Complex sample weights were used to obtain nationally representative estimates

**Table 2:** Mortality due to primary brain tumours in China, 2013 (1/100,000)

	Men				Women				Total	
Age group	Population	Cases	Mortality*	95% CI*	Population	Cases	Mortality*	95% CI*	Mortality*	95% CI*
0~	45,548	0	--	--	39,914	0	--	--	--	--
15~	41,209	0	--	--	40,169	1	0.5	0.06-3.3	0.2	0.03-1.6
25~	44,351	0	--	--	45,246	0	--	--	--	--
35~	52,561	0	--	--	50,438	0	--	--	--	--
45~	45,289	0	--	--	45,378	1	1.4	0.2–10.4	0.7	0.1–5.1
55~	38,189	1	0.3	0.04–2.2	39,886	2	7.5	1.2–46.7	3.9	0.7–22.5
65~	21,283	4	26.6	8.4–83.5	21,962	2	5.9	1.5–23.9	16.3	6.2–42.9
75~	11,295	2	15.1	3.1–73.8	12,993	2	2.8	0.4–18.0	8.2	2.2–31.4
Total	299,725	7	1.8	0.7–4.6	295,986	8	1.5	0.5–4.5	1.6	0.8–3.3
Subtotal (≥15)	254,177	7	2.2	0.9–5.6	256,072	8	1.8	0.6–5.3	2.0	1.0–4.0
Death No.	--	--	11,878.8	4640.8–30,405.4	--	--	9336.5	3058.9–28,496.9	21,215.3	10,427.2–43,164.6

*Annual mortality between 1 Sept 2012 and 31 Aug 2013; mortalities and 95% CIs were estimated with individual final weights

**Table 3:** Detection rate of primary brain tumours in participants with suspected symptoms (/signs) (1/100,000)

	Men				Women				Total	
Age group	Population	Cases	Detection rate*	95% CI*	Population	Cases	Detection rate*	95% CI*	Detection rate*	95% CI*
0~	19	0	--	--	9	0	--	--	--	--
15~	20	0	--	--	14	2	14,285.7	1730.1–51,604.9	5882.4	712.4–21,249.1
25~	66	0	--	--	77	0	--	--	--	--
35~	244	1	409.8	10.4–2283.5	316	6	1898.7	696.8–4132.7	1250.0	502.6–2575.5
45~	895	3	335.2	69.1–979.6	1022	6	587.1	215.4–1277.8	469.5	214.7–891.2
55~	2270	3	132.2	27.3–386.2	2497	6	240.3	88.2–523.0	188.8	86.3–358.4
65~	2234	7	313.3	126.0–645.6	2356	8	339.6	146.6–669.1	326.8	182.9–539.0
75~	1548	3	193.8	40.0–566.4	1403	1	71.3	1.8–397.1	135.5	36.9–347.1
Total	7296	17	233.0	135.7–373.1	7694	29	376.9	252.4–541.3	306.9	224.7–409.3

*Detection rates and 95% CIs were estimated based on the Poisson distribution

### Nationwide mortality due to PBTs in China

In China, the weighted mortality due to PBT was 1.6 (95% CI 0.8–3.3) per 100,000 population, 1.8 (95% CI 0.7–4.6) per 100,000 population in men, and 1.5 (95% CI 0.5–4.5) per 100,000 population in women (see Table 2); 1.2 (95% CI 0.4–3.6) per 100,000 population in urban residents and 2.1 (95% CI 0.9–5.4) per 100,000 population in rural residents; and 0.5 (95% CI 0.1–2.2) per 100,000 among eastern Chinese, 2.4 (95% CI 1.0–6.0) per 100,000 population in central Chinese, and 2.4 (95% CI 0.6–9.0) per 100,000 population in western Chinese. According to the above-estimated mortality rates, there were 21,215 (95% CI 10,427–43,165) deaths annually from PBTs in the population, with 11,879 (95% CI 4641–30,405) deaths in men and 9337 (95% CI 3059–28,497) deaths in women in China (see Table 2).

### Detection rate of PBTs among people with suspected symptoms (/signs) and influencing factors

Among the 14,990 people with suspected symptoms (/signs), the PBT detection rate was 306.9 (95% CI 224.7–409.3) per 100,000 population, 233.0 (95% CI 135.7–373.1) per 100,000 population in men, and 376.9 (95% CI 252.4–541.3) per 100,000 population in women (see Table 3); 262.2 (95% CI 160.2–404.9) per 100,000 population in urban residents and 353.2 (95% CI 230.7–517.5) per 100,000 population in rural residents; and 222.2 (95% CI 110.9–397.6) per 100,000 population in eastern Chinese, 367.6 (95% CI 240.1–538.6) per 100,000 population in central Chinese, and 303.3 (95% CI 138.7–575.8) per 100,000 population in western Chinese.

The factors influencing detection rates included age, gait disturbance, visual anomalies, dizziness/vertigo, and headache. The detection rate was higher in people aged 35–44 years than in people aged ≥75 years (OR 5.52; 95% CI 1.37–22.16; *P*=0.016), in people with an unsteady gait than in people without an unsteady gait (2.46; 1.09–5.51; *P*=0.029), in people with visual anomalies than in people without visual anomalies (3.84; 1.88–7.85; *P*<0.001), and in people with headache than in people without headache (2.06; 1.10–3.86; *P*=0.023). However, the detection rate was lower in people with dizziness/vertigo than in people without dizziness/vertigo (0.45; 0.23–0.87; *P*=0.017) (Table 4).

**Table 4:** Factors influencing detection rates in participants with suspected symptoms (/signs) of primary brain tumour

Factors	Participants	PBT No.	%	Adjusted^a^ OR	95%CI	*P* value
Age group, *n* (%)						
0~	28	0	--	--	--	0.998
15~	34	2	5.9%	13.42	0.89–202.85	0.061
25~	143	0	--	--	--	0.996
35~	560	7	1.3%	5.52	1.37–22.16	0.016
45~	1917	9	0.5%	2.32	0.64–8.48	0.203
55~	4767	9	0.2%	0.95	0.27–3.29	0.933
65~	4590	15	0.3%	1.78	0.57–5.61	0.322
75~	2951	4	0.1%	Reference	Reference	
Sex, *n* (%)						
Men	7296	17	0.2%	0.65	0.34–1.23	0.186
Women	7694	29	0.4%	Reference	Reference	
Ethnicity, *n* (%)						
Han	13,756	39	0.3%	Reference	Reference	
Other	1234	7	0.6%	1.02	0.35–2.95	0.966
Education, *n* (%)						
Primary school	9176	21	0.2%	Reference	Reference	
Middle school	5297	22	0.4%	1.49	0.75–2.94	0.253
College and higher	504	0	--	--	--	0.993
Missing	13	3	23.1%	30.07	0.09–9575.23	0.247
Marital status, *n* (%)						
Married	12,059	36	0.3%	Reference	Reference	
Single	238	3	1.3%	2.96	0.51–17.40	0.229
Widowed	2640	4	0.2%	0.60	0.20–1.78	0.361
Other	33	0	--	--	--	0.998
Missing	20	3	15.0%	2.03	0.01–593.19	0.808
Occupation, *n* (%)						
Students	42	1	2.4%	0.99	0.04–22.32	0.993
Worker	620	1	0.2%	0.44	0.06–3.47	0.436
Farmer	8343	25	0.3%	Reference	Reference	
Employee	350	1	0.3%	1.05	0.13–8.44	0.960
Entrepreneur	393	3	0.8%	1.45	0.38–5.45	0.585
Retiree or homemaker	5154	12	0.2%	1.01	0.44–2.36	0.974
Other	76	0	--	--	--	0.997
Missing	12	3	25.0%	5.90	0.02–1868.59	0.546
Place of residence, *n* (%)						
Urban	7628	20	0.3%	0.72	0.35–1.46	0.359
Rural	7362	26	0.4%	Reference	Reference	
Geographic location, *n* (%)						
Eastern China	4950	11	0.2%	0.97	0.38–2.48	0.946
Central China	7073	26	0.4%	1.93	0.85–4.40	0.119
Western China	2967	9	0.3%	Reference	Reference	
Symptoms (/signs), *n* (%)						
Conscious disturbance						
Yes	1881	3	0.2%	0.42	0.12–1.44	0.167
No	13,109	43	0.3%	Reference	Reference	
Limb paralysis or weakness						
Yes	5842	13	0.2%	0.74	0.32–1.72	0.480
No	9148	33	0.4%	Reference	Reference	
Facial paralysis						
Yes	2654	2	0.1%	0.26	0.06–1.18	0.080
No	12,336	44	0.4%	Reference	Reference	
Unsteady gait						
Yes	4180	14	0.3%	2.46	1.09–5.51	0.029
No	10,810	32	0.3%	Reference	Reference	
Speech disorders						
Yes	3525	6	0.2%	0.71	0.27–1.87	0.491
No	11,465	40	0.3%	Reference	Reference	
Paraesthesia						
Yes	3594	8	0.2%	0.72	0.32–1.64	0.437
No	11,396	38	0.3%	Reference	Reference	
Visual anomalies						
Yes	1777	12	0.7%	3.84	1.88–7.85	<0.001
No	13,213	34	0.3%	Reference	Reference	
Dizziness/vertigo						
Yes	7521	17	0.2%	0.45	0.23–0.87	0.017
No	7469	29	0.4%	Reference	Reference	
Headache						
Yes	4503	22	0.5%	2.06	1.10–3.86	0.023
No	10,487	24	0.2%	Reference	Reference	
Nausea/vomiting						
Yes	2766	7	0.3%	0.66	0.27–1.59	0.351
No	12,224	39	0.3%	Reference	Reference	

^a^All other variables in the table were adjusted for each interesting variable in a multivariate logistics regression model

## Discussion

### Mortality due to PBT

In this survey, the weighted mortality due to PBT was 1.6/100,000 population, 1.8/100,000 population in men, and 1.5/100,000 population in women, excluding tumours originating from spinal cord and brain metastases from other systems outside the central nervous system. The corresponding mortalities were lower than 3.96/100,000 population (4.30/100,000 population in males and 3.60/100,000 population in females) for brain and CNS tumours, according to the National Central Cancer Registry of China [12], and lower than 2.8 per 100,000 population in males and 2.0 per 100,000 population in females according to annual, global, age-standardized mortality due to primary malignant brain tumours [13]. We speculate that the reason for the difference is most likely attributed to different study designs and diagnostic criteria, although the real cause for the difference is unknown.

Generally, mortality due to PBT is higher in developed regions than in developing regions [13]. However, mortality due to PBT in urban residents was lower than that in rural residents in this survey. Mortality due to PBT in eastern Chinese individuals in developed areas was also lower than those in central and western Chinese individuals in intermediately and underdeveloped areas. Mortality due to intracranial tumours in 21 rural areas was 4.1/100,000 [11], which was higher than 2/100,000 in six cities [10] during the same period. Data from the National Central Cancer Registry of China were similar; the age-standardized mortality rate in rural areas was 3.33/100,000, which was higher than 2.77/100,000 in urban areas [12]. The true reasons for the contrasting results, as well as differences in detection methods and bias due to the sampling survey method, need to be further explored. Furthermore, mortality due to PBT in China has not increased over the past 30 years according to the analysis of existing studies.

### Implication of an improved detection rate based on PBT symptom screening

Despite the relatively low prevalence of PBTs in the population, the detection rate of PBTs could be greatly improved by PBT symptom screening (for an unsteady gait, visual anomalies, headache, etc.) according to the data from the survey. Interestingly, patients with dizziness/vertigo were less likely to have brain tumours than those without dizziness/vertigo. This indicates that most brain tumours originating from regions other than the cerebellum or posterior circulation territories did not induce symptoms of dizziness/vertigo. After reviewing the relevant literature, we could not find any information to perform a comparative analysis. The detection rate of brain tumours in the group aged 35–44 years was higher than that in the group aged 75 + years; this may be due to different responses to brain tumour symptoms, leading to incidental discovery. Table 5 lists the study designs and findings of previous PBT prevalence surveys in China and other regions worldwide. In China, three studies have estimated the prevalence of brain tumours; the prevalence rates were 32 (20.91–43.09)/100,000 population in six cities in 1985 [10], 6.9 (1.75–12.05)/100,000 population in 21 rural areas in 1989 [11], and 24.56 (14.85–34.27)/100,000 population in five cities in 2011 [14]. Further, the prevalence rates from the three previous studies were obviously lower than 130.8/100,000, 209.0/100,000, and 221.8/100,000 population as reported in the USA [2, 3]; 68/100,000 population in males and 93/100,000 population in females in Sweden [15]; 94/100,000 population for pituitary adenoma in Belgium [16]; and 77.6/100,000 population for pituitary adenoma in England [17]. The results were similar to the 5-year partial prevalence of brain and other CNS tumours at 13.8/100,000 population in males and 15.9/100,000 population in females in France [18], and 26.3/100,000 population for CNS glial tumour and 4.7/100,000 population for CNS nonglial and pineal gland tumours in Europe [4]. We note that these are marked differences in methods and results across different studies on the prevalence of PBTs. Both higher detection and survival rates in developed countries and regions might partially explain why the prevalence rates of primary brain tumours and pituitary adenomas are so higher than those in China.

**Table 5:** Comparison of designs and findings of previous primary brain tumour prevalence surveys

Study, date, reference	Region	Population	Prevalence date	Design	Diagnosis	Prevalence (1/100,000)	Subgroup/subtype (1/100,000)
Wang, 1985 [10]	China	All ages; 65,067 in urban areas including six cities in China	Jan 1, 1984	Cross-sectional survey, point prevalence	Clinical diagnosis; histology; imaging	32 (20.91–43.09)	--
Li, 1989 [11]	China	All ages; 246,812 people in rural areas of 21 provinces in China	Jan 1,1985	Cross-sectional survey, point prevalence	Clinical diagnosis; histology; imaging	6.9 (1.75–12.05)	Men, 7.3; women, 6.4
Jiang, 2011 [14]	China	All ages; 2,589,448 people in five cities in China	2006	Registry of primary brain tumours, period prevalence	Clinical diagnosis; histology; imaging	24.56 (14.85–34.27)	Men, 18.84 (10.33–27.35); women, 30.57 (19.73–41.41)
Davis, 2001 [2]	USA	All ages; no data on population	2000	Prevalence rates were estimated using age-specific incidence rates (1985–1994) and survival curves from two population-based tumour registries from the Central Brain Tumor Registry of the United States in a statistical model.	Behavior codes; histology	130.8	Benign, 97.5; malignant, 29.5 (men, 32.7; women, 25.9)
Porter, 2010 [3]	USA	All ages; no data on population	2004	An estimate of the prevalence of disease in the United States based on incidence data for 2004 and survival data for 1985–2005 obtained by the Central Brain Tumor Registry of the United States from selected regions for the year 2004.	Histology	209	Men, 158.7; women, 264.8; glioma, 34.3
Porter, 2010 [3]	USA	All ages; no data on population	2010	A projected estimate of the prevalence of disease in the United States based on incidence data for 2004 and survival data for 1985–2005 obtained by the Central Brain Tumor Registry of the United States from selected regions for the year 2010.	Histology	221.8	--
Crocetti, 2012 [4]	Europe	All ages; 2008 European population (497,455,033)	Jan 1, 2003	Point prevalence at the index date estimated based on incidence and follow-up data from 22 population-based cancer registries;	Cancer registries coded according to ICD-O-3	--	Glial tumour of CNS, 26.3; non-glial tumour of CNS and pineal gland, 4.7
Adami, 1989 [15]	Sweden	All ages; 8.3 million inhabitants constituting the Swedish population	Dec 31, 1984	National Swedish Cancer Registry, point prevalence	Clinical diagnosis; histology	--	Men, 68; women, 93
Daly, 2006 [16]	Belgium	All ages; 71972 inhabitants in Belgium	Sep 30, 2005	Cross-sectional hospital-based clinic review, point prevalence	Clinical diagnosis; histology; hormonal workup	--	Pituitary adenoma, 94 (72.2–115.8)
Fernandez, 2010 [17]	Banbury, Oxfordshire, England	All ages; 81149 inhabitants in Banbury	Jul 31, 2006	Cross-sectional administrative database, point prevalence	Clinical diagnosis; histology; hormonal workup	--	Pituitary adenoma, 77.6
Colonna, 2008 [18]	France	All ages; whole French population; no data on population	1998–2002	Incidence and survival data from French cancer registries were used to estimate specific 5-year partial prevalence rates	Cancer registries coded according to ICD-O-3	--	Men, 13.8; women, 15.9

It should be noted that the importance of PBT detection based on symptom screening is the potential feasibility of investigating the prevalence of PBTs in populations in the future using a two-step method including symptom screening and neurologist review. Moreover, the same method of PBT case ascertainment in the population allows for comparisons of PBT prevalence rates among different populations.

### Sex difference in the epidemiology of PBTs

In both sexes, the incidence of and mortality due to primary benign and malignant brain tumours differs, although no sex differences in mortality and detection were found in this survey. The incidence of and mortality due to malignant brain tumours are generally higher in males than in females. The annual, global, age-standardized incidence of primary malignant brain tumours is 3.7/100,000 population in men and 2.6/100,000 population in women [13, 14]. The annual, global, age-standardized mortality due to primary malignant brain tumours is 2.8/100,000 population in men and 2.0/100,000 population in women [13]. Malignant gliomas are more common in males than in females, with a male-to-female ratio ranging from 1.5:1 to 2.2:1, whereas benign meningiomas are more common in females than in males, with a male-to-female ratio ranging from 0.5:1 to 0.9:1 [1, 19]. Gliomas affect approximately 40% more males than females, and meningiomas affect approximately 80% more females than males [20]. A higher incidence of primary glioblastoma (GBM) has been reported in men than in women; however, this is not true for secondary GBM [21]. As mentioned above, the sexual heterogeneity of different types of PBTs may be observed across different populations.

Previous studies have found that men have a higher prevalence of primary malignant brain tumours than women [2, 11]. In contrast, most previous studies confirmed that the prevalence of PBTs in women was higher than that in men [3, 14, 15, 18]. The exclusion of benign brain tumours in early studies may explain why the prevalence of brain tumours in males is relatively higher than that in females. To some extent, this explains why the prevalence of PBTs in women is higher than that in men in most studies.

### Strengths and limitations

This survey was a cross-sectional survey, with representativeness of the Chinese population, but it also had many shortcomings. Symptoms such as headache, vomiting, motor and sensory dysfunction, limb weakness, paralysis and numbness, visual impairment, visual field defect, language disorder, and imbalance may help to identify cases; however, a small number of patients with brain tumours may lack specific symptoms. In this study, we did not analyse other symptoms that may be associated with brain tumours, such as convulsions or seizures, olfactory disorders, nervous deafness, mental decline, psychiatric symptoms, endocrine disorders, and developmental abnormalities. Over half of the confirmed brain tumours lacked pathological typing. Forty-two percent of patients with suspected symptoms during preliminary screening who needed further review and diagnosis by a neurologist did not have any CT/MRI data. In addition, symptomatic retrospective bias is inevitable. The incidence of brain tumours in children is generally lower than that in adults, and no brain tumours in children were found in this survey, indicating that the symptom screening method is not suitable for epidemiological investigations of brain tumours in children. Nonetheless, we believe that the findings of this survey accurately reflect the rates in people over 15 years old.

## Conclusions

In summary, this survey accurately represented for mortality due to PBTs in China. It is estimated that 21,215 (10,427–43,165) deaths from primary brain tumour occur annually in China. Although the incidence and prevalence of PBTs were relatively low in the population, the detection rate of PBTs can be greatly improved by symptom screening. These findings could provide a data reference for relevant health administrative departments or professional associations tasked with health care policy making or disease management in patients with brain tumours.

## Data Availability

The manuscript does not refer to any new software, application, or tool. The authors do not wish to share data analysed in this manuscript as no such consent was provided by the investigated participants and no approval of the Bioethics Committee was obtained.

## Abbreviations

PBTs: Primary brain tumours
PPS: Probability proportionate to population size
DSPs: Disease surveillance points
CDC: Center for Disease Control and Prevention
ICD-10-CM: International Classification of Diseases, Tenth Revision, Clinical Modification
CT: Computed tomography
MRI: Magnetic resonance imaging
CIs: Confidence intervals
